# Attitude and performance in controlling dust particles from dental prosthesis and appliance adjustment: a survey of dentists and dental assistants

**DOI:** 10.1038/s41405-024-00206-7

**Published:** 2024-03-20

**Authors:** Nareudee Limpuangthip, Prarom Salimee, Phanomporn Vanichanon

**Affiliations:** 1 Department of Prosthodontics, Faculty of Dentistry, Chulalongkorn University, Bangkok, 10330 Thailand; 2 Department of Occlusion, Faculty of Dentistry, Chulalongkorn University, Bangkok, 10330 Thailand

**Keywords:** Health care, Occupational health

## Abstract

**Objectives:**

To assess the attitude and practices of dentists and dental assistants in managing dust particles generated during dental prostheses or appliances grinding and polishing.

**Materials and methods:**

Data were collected from 207 dentists and 125 dental assistants through an online questionnaire. The questionnaire included adjusted prosthesis types, self-protective methods, types and frequency of protective device use, and reasons for non-frequent use.

**Results:**

Protective grinding devices, including dust protective boxes and mounted plastic sheets, were commonly used for substantial acrylic resin adjustments, while air blowing was preferred for minor adjustments. Post-COVID-19, there was a 3-fold increase in the use of protective grinding devices among dentists and a 1.3-fold increase among dental assistants. During try-in procedures, dentists commonly rinsed prostheses with water rather than using disinfectants. Non-frequent users adopted self-protection methods, such as face shields and air filters. Surgical drapes and high-volume evacuators were used for patient’s protection.

**Conclusion:**

Despite an increased tendency of the use of protective grinding devices following COVID-19, a significant number still report infrequent use. Identified protective devices in this study have drawbacks not fully meeting dentists’ expectations. Invention of a more user-friendly device is necessary to ensure regular use, preventing potential toxicity from dust particles.

## Introduction

Air pollution is a significant concern due to its adverse health effects, including respiratory and cardiovascular diseases, as well as liver and renal function [1, 2]. In the healthcare setting, maintaining indoor air quality is essential for infection control, safeguarding healthcare workers and patients [3]. The dental working environment contributes to air pollution, primarily arising from aerosols generated during intraoral procedures and extraoral dental prosthesis or appliance grinding [4–6]. Airborne dental material dust poses health risks to dental healthcare workers and patients, potentially damaging the respiratory system by lodging in the middle airway or penetrating and damaging human alveoli [4, 7]. Furthermore, it increases the risk of infectious disease transmission, such as tuberculosis and severe acute respiratory syndrome, through droplets and aerosol inhalation. This issue has become of increased concern in dental offices, especially in prosthodontic practices, particularly during the COVID-19 pandemic era [8, 9].

Numerous protective protocols have been proposed to minimize the release of particles during intraoral dental procedures, involving the use of both high-speed and low-speed hand pieces. These protective protocols encompass various strategies, including self-protection wear, intraoral and extraoral devices, disinfection methods, and external machines such as high-efficiency particulate absorbing filter or air purifiers [5, 9, 10]. Self-protection involves personal protective equipment, masks, and face shields, while extraoral devices encompass high-volume evacuators and extraoral suction. In addition to airborne droplets, dental appliances and materials release dust particles during extraoral grinding and adjusting procedures, e.g., composite resin, acrylic resin, metal, porcelain, and ceramics [11–13]. The dust particles originating from dental material grinding exhibit different morphologies, particle sizes, and elemental compositions, leading to varying toxicity levels [4, 7].

To control the dispersion of dust particles spreading from extraoral dental prosthesis grinding, several tools and equipment have been employed to minimize dust dispersal during dental procedures and prosthesis grinding. These include high-volume evacuators, high-power suction [11, 14], and extraoral suction devices. However, high-power suction may not effectively minimize dust dispersion. Moreover, the availability of extraoral suction devices is typically limited to private hospitals and seldom available in small-scale dental clinics, public hospitals, and dental schools due to their relatively high cost and space requirements within dental treatment rooms. Many clinical settings rely on portable high-powered suction, some of which are integrated with dental units to prevent aerosol and dust particle dispersion. Furthermore, various commercially available and self-made grinding tools, such as plastic dust-collecting boxes and mounted plastic sheets, are used for dental prosthesis grinding and polishing in dental laboratories and clinics. However, there is a lack of compulsory rules or standard guidelines for managing infection control and dust particle dispersion during grinding and polishing procedures. The objective of this cross-sectional survey was to assess the awareness and practices of dentists and dental assistants in managing dust generated during dental prosthesis and appliance grinding and polishing.

## Materials and methods

The present study employed a cross-sectional study design conducted from January to June 2022. The eligible participants were Thai dentists actively engaged in dental practice involving grinding any type of dental appliances or prostheses, as well as dental assistants regularly assisting in dental procedures that involved grinding or polishing. Individuals who declined to provide information were excluded from the study. The study protocol was approved by the Human Research Committee of the Faculty of Dentistry, Chulalongkorn University (HREC-DCU 2021-077) and was conducted in accordance with the principles of the Declaration of Helsinki. Prior to responding to the questions, all participants provided electronic written consents. Informed consent was obtained from all participants prior to enrolling the study.

G-power software (version 3.1.9.4) was used to determine the required sample size of the study. Our aim was to investigate the difference in the percentage of participants who currently used protective grinding devices before and after the COVID-19 pandemic. The inequality proportion of two dependent samples was used as the statistical test. To inform the sample size estimation, a pilot study was conducted among 30 dentists and 30 dental assistants. The pilot study revealed that the odds of using a grinding box after the pandemic were 3-fold higher compared with before COVID-19. Based on 0.25 discordant pairs, a power level of 0.8, and an alpha (α) error of 0.05, a minimum sample size of 120 participants in each group was required to obtain statistically meaningful results.

### Questionnaire design and contents

The questionnaire and its contents were developed from a combination of research inquiries and an extensive review of the relevant literature. The investigators identified pivotal questions related to the study’s topic. Potential questions were selected after thorough group discussion and evaluated to ascertain their suitability for inclusion. The criteria for selection were based on their comprehensibility, accuracy, and relevance to the topic [15]. To ensure content validity of the questionnaire, a panel of experts comprising two prosthodontists, one orthodontist, two general dentists, and two dental assistants with a minimum of 10-year clinical experience, assessed the questions. Consensus on any disagreement was achieved through group discussion. To assess face validity, the questionnaires were presented to 10 dentists and 10 dental assistants familiar with dental prosthesis grinding and the use of protective grinding devices. Any issues identified were resolved by modifying the questionnaires accordingly. To prevent participant fatigue and potential drop-outs, the questionnaire was designed to be completed within an estimated time frame of 5–10 min.

The questionnaire comprised three sections:

Section 1: Demographic information. This section gathered demographic data, including age, sex, educational level, type of educational institutes, field of dental specialty or an assistant, years of clinical experience, and their typical grinding position in clinical practice.

**Fig. 1:**
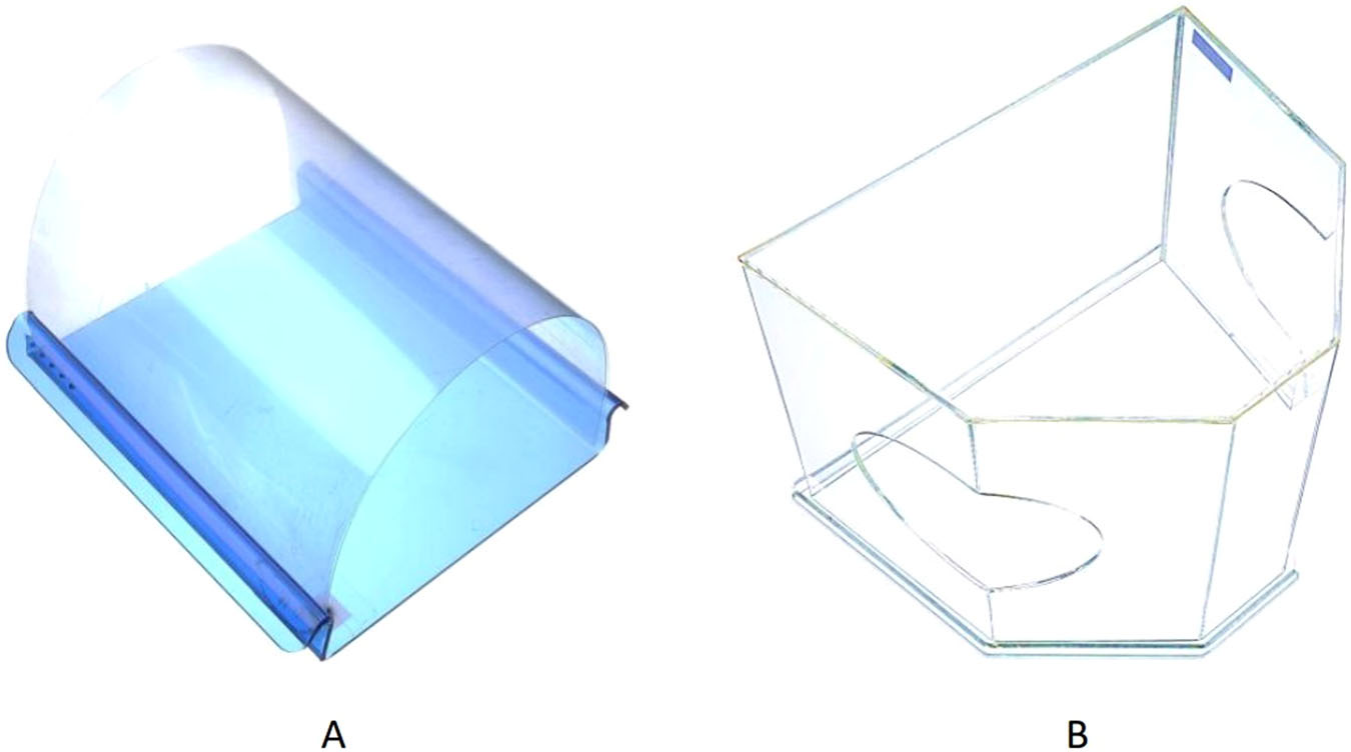
Two protective grinding devices publicized in the questionnaire. **A** Mounted plastic sheet (occlu dust guard™, designed by Dr. Phanomporn Vanichanon, consists of a disposable clear plastic sheet mounted on a solid acrylic base); **B** Dust protective box.

Section 2: Preventive methods, protective devices, and their frequency of use for dental prosthesis and appliance adjustment. The participants were asked about the preventive methods and protective devices used during dental prosthesis and appliance grinding. The questions were based on the stage of prosthesis use, as well as the quantity and size of ground particles. The amount of prosthesis grinding was determined by factors, such as volume and time. Moreover, the participants were asked about the frequency of using protective grinding devices before and after the onset of the COVID-19 pandemic. Two commonly used protective grinding devices available in Thailand are mounted plastic sheets and dust protective boxes and (Fig. 1A, B). Responses were rated on a five-level ordinal scale; never, rarely (1 to <10% of the work), sometimes (10 to <40%), often (40 to <70%), and very often (70 to 100%). Based on the frequency of use, the participants were categorized as frequent users (often to very often) or non-frequent users (sometimes to never).

Section 3: This section comprised different questions for frequent and non-frequent users. This section varied depending on whether the participants were frequent or non-frequent users of grinding devices. Frequent-users were asked about the specific types of protective grinding devices they used, their cleaning methods after use, their attitudes towards each type of device, and any issue they encountered while using them. In contrast, non-frequent users were asked about their self-protection methods, particle management in the dental treatment room, particle management after each patient, and the reasons for infrequent use.

### Questionnaire distribution

An online survey was created using Google Forms® tool and distributed to Thai dentists and dental assistants through professional social media platforms, which were Line and Facebook. The collected data were anonymized and exported using Google Forms® to a Microsoft Excel® spreadsheet (Microsoft Corporation, Redmond, WA). Subsequently, the data were securely stored separately, ensuring that the responses could not be traced back to the individual participants.

### Data analysis

The data were analyzed using the statistical software package SPSS version 28.0. Descriptive statistics was used as an analyzing tool for quantitative data calculation including the mean, standard deviation (SD) and percentage distribution.

## Results

Initially, 208 dentists and 126 dental assistants participated in the survey. However, one individual from each profession refused to participate in the study. The characteristics of the 207 dentists and 125 dental assistants who completed the questionnaire are presented in Table 1. The dentists had a mean age (SD) of 42 ± 15.5 years old (range 24–65 years old), while the dental assistants had a mean age (SD) of 46.3 ± 16.4 years old (range 19–60 years old). Most of them reported their preferred grinding positions as being either besides or at the back of the patients.

**Table 1:** Participant characteristics.

Variables	Dentists (*N* = 207)		Dental assistants (*N* = 125)	
	*N*	%	*N*	%
Sex: Male	67	32.4	13	89.6
Female	140	67.6	112	10.4
Educational level				
Lower than bachelor	0	0.0	80	64.0
Bachelor	98	47.3	44	35.2
Higher than bachelor	109	52.7	1	0.8
Dental specialist/assisting (3 most frequent)				
Prosthodontist	99	47.8	52	41.6
Operative dentistry specialist	8	3.9	87	69.6
Orthodontist	9	4.3	2	1.6
Occlusion & Orofacial pain	2	1.0	7	5.6
Maxillofacial surgeon specialist	6	2.9	4	3.2
General practitioners	67	32.4	84	67.2
Others (periodontics, endodontics, pedodontics)	16	7.7	15	12.0
Clinical experience (years)				
<5	111	54.1	75	60.0
5 to <10	43	20.8	19	15.2
10 and above	52	25.1	31	24.8
Preferred grinding positions				
Besides or behind the patients	98	47.3	N/A	
On the dentist’s lap	56	27.1		
On the dental unit	35	16.9		
At the counter	116	7.7		
On mobile tray unit	2	1.0		

N/A, not applicable.

Dentists and dental assistants reported similar use of protective grinding devices and infection control methods during prosthesis grinding, except in the case of lab as-received prostheses, where dentists infrequently employed protective grinding devices (Table 2). Although the dental assistants frequently prepared protective grinding devices, the dentists often opted not to use any devices but instead used air blown from a triple syringe. They also preferred using disinfectant or soap for prosthesis cleaning. Protective grinding devices and disinfectant were mostly used when working on worn prostheses or with a substantial amount of acrylic resin. In contrast, for minor grinding and polishing, only blown air was used. During the try-in process, the dentists more commonly rinsed the prosthesis with water rather than using disinfectant or soap.

**Table 2:** Appliances and their frequency of use, and disinfection methods for dental prosthesis grinding (*N* for dentists (D) = 207, dental assistants (A) = 125).

Types of dental prostheses	Respondent	Preventive devices/methods used/prepare:			Methods of infection control during grinding			
		None/Air syringe	High power suction	Grinding devices	No	Water rinsing	Soap cleaning	Disinfectant
Based on stage of usage								
Lab as-received prostheses	D	59.9	15.5	24.6	13.6	22.7	21.7	42.0
	A	18.4	21.6	60.0	8.0	32.8	13.6	45.6
Previously worn prostheses	D	20.8	27.5	51.7	5.3	27.1	19.8	47.8
	A	10.4	24.0	65.6	0.8	18.4	26.4	54.4
Suspected or confirmed infected patients	D	15.0	17.4	65.7	4.8	4.8	7.7	80.2
(*n* for D = 203, A = 120)	A	6.4	22.4	67.2	0.8	4.8	9.6	80.8
During first visit try-in and insertion	D	26.1	25.6	48.3	11.1	46.4	15.5	27.1
	A	11.2	26.4	62.4	1.6	40.8	17.6	40.0
Based on amount and size of the ground particles								
Substantial quantity of acrylic resin^a^	D	26.1	28.5	45.4	N/A			
	A	6.4	24.8	86.6				
Small quantity of acrylic resin	D	48.8	27.5	23.7				
	A	28.0	44.0	28.0				
Permanent fixed restorations	D	55.1	30.4	14.5				
	A	25.6	36.8	37.6				
Final polishing, Glossing	D	57.5	23.7	18.8				
	A	24.8	35.2	40.0				

N/A, not appliable.

^a^Substantial quantity included grinding removable dentures, baseplate, occlusal splint, surgical stent, removable orthodontic appliances. Small quantity included temporary crown and bridge. Permanent fixed restoration included composite resin, all ceramic, porcelain, and metal-based fixed prostheses.

**Fig. 2:**
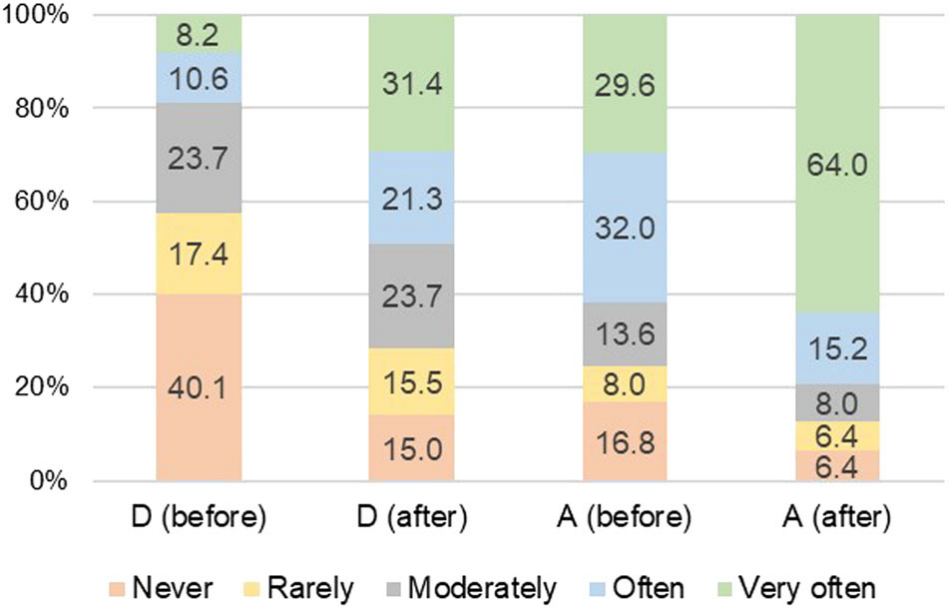
Distribution (%) of dentists and dental assistants. Percentage of dentists (D) and dental assistants (A) with various frequencies of grinding device used during before and after COVID-19 pandemic.

**Fig. 3:**
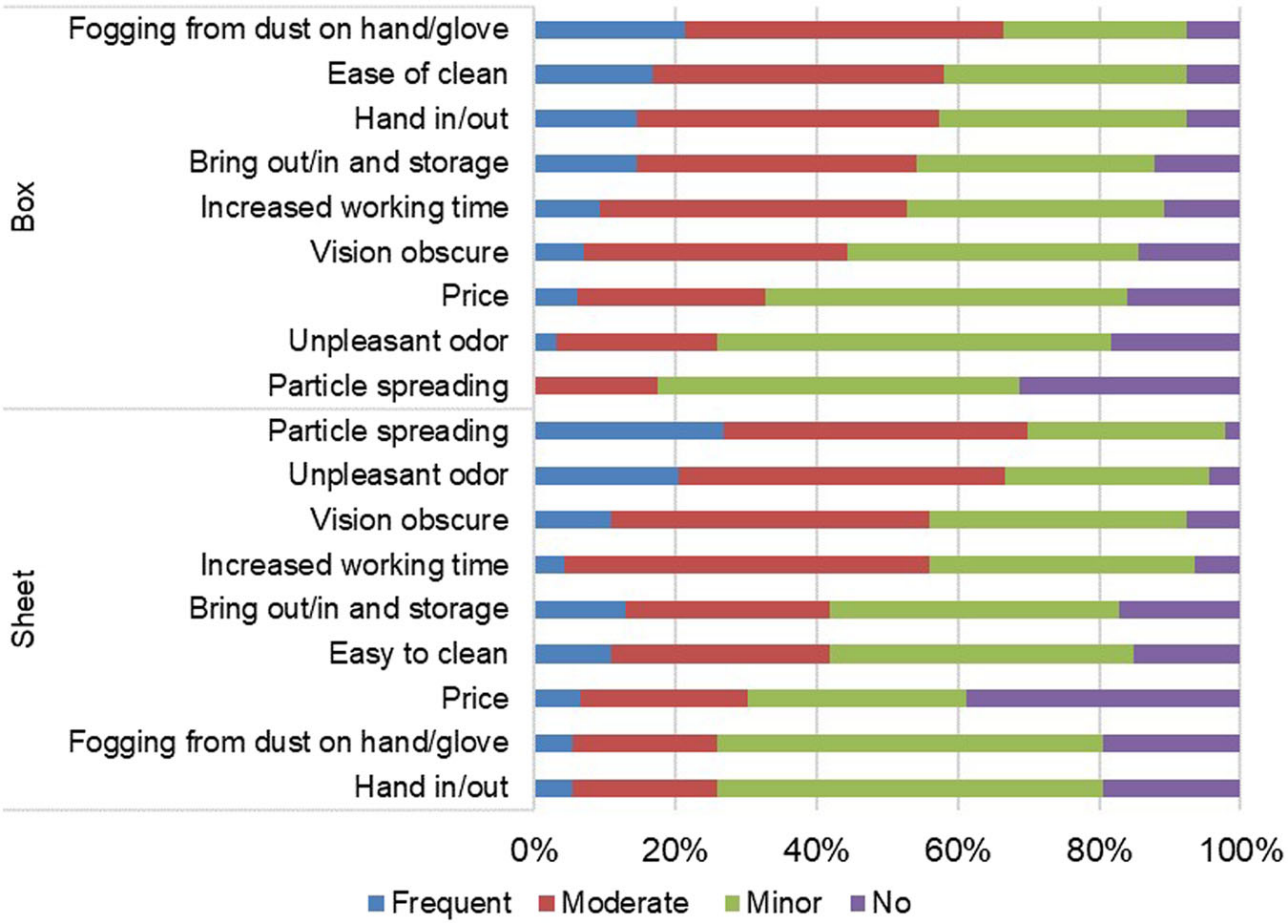
Distribution (%) of frequent-user dentists of protective grinding devices. Percentage of the frequent-user dentists who reported problems related to a protective box and mounted plastic sheet used during grinding and polishing (*n* = 131).

Following the COVID-19 pandemic, the use of protective grinding devices significantly increased by approximately 3-fold among dentists and 1.3-fold among dental assistants (Fig. 2). Among frequent users (76.4% of dentists and 87.2% of dental assistant), a dust protective box was the most commonly used device, followed by a mounted plastic sheet (Table 3). However, various problems were reported, such as obscured vision and increased working time (Fig. 3). For non-frequent users for both dental professions, self-protection included the use of N95 masks or their equivalents, face shields, and air filters (Table 4). To protect the patients, the use of surgical drapes to cover the patients’ face and upper body, and using a high-volume evacuator were reported. The reasons for not using protective grinding devices included their unavailability, concerns about work delays, and the perception of their being cumbersome to use.

**Table 3:** Frequently used grinding devices and their experiences in frequent users.

Topics	Dentist (*n* = 144)	Dental assistant (*n* = 109)
Type of device (more than 1 item)		
Dust protective box	111 (77.1)	90 (82.6)
Mounted plastic sheet	53 (36.8)	45 (41.3)
Plastic bag	13 (9.0)	4 (3.7)
Self-adapted paper or plastic box, pipe	9 (6.3)	5 (4.6)
Device cleaning after each case		
No	29 (20.1)	5 (4.6)
Yes	115 (79.9)	104 (95.4)

**Table 4:** Protection methods and reasons for never/rarely using grinding appliances in non-frequent users.

Topics	Options	Dentists (%) (*n* = 63)	Dental assistants (%) (*n* = 16)
Self-protection method	N95 mask or equivalent	79.4	93.8
(up to 3 answer)	Glasses	25.4	50.0
	Face shield	95.2	93.8
	Extraoral dental suction/ High volume evacuator (included dental aerosol suction system (easy prep®))	14.3	68.8
	Air filters	71.4	75.0
	Negative pressure room	1.6	12.5
Particle management	None, floor cleaning	77.8	56.2
(up to 3 answer)	Surgical drape	36.5	56.2
	High volume evacuator	41.3	93.8
Particle management after each patient (room cleaning)	No	28.6	6.2
	Yes	71.4	93.8
Reason for rarely use	Not available	50.8	50.0
(up to 3 answer)	Delay the work	41.3	12.5
	Cumbersome	28.6	18.8
	Easier to clean the floor after work	15.9	25.0
	Difficult to clean device	15.9	0.0
	Not related to the work	14.3	25.0
	No idea to use/Not request by dentist	N/A	37.5
	No favorite device	22.2	N/A
	Vision obscure	3.2	N/A

## Discussion

The findings of this survey demonstrated a notable increase in the use of protective grinding devices by dentists and dental assistants following the COVID-19 pandemic. The results also highlighted that a considerable number of Thai dentists and dental assistants had awareness of using protective grinding devices and protocols to mitigate the generation of dust particles during dental prosthesis grinding and adjustment. However, the actual implementation of effective particle control methods exhibited variability among dentists and dental assistants.

Various protocols for controlling dust particles and infection are used based on the stage of dental prosthesis use, as well as the quantity and size of the ground particles. Our results indicated that dentists frequently employed protective grinding devices and disinfectants when working on worn prostheses, especially those worn by infected patients. In the case of lab as-received prostheses and those worn by infected patients, dentists often cleaned the prostheses with disinfectant or soap, and prevented particle dispersion through air blowing or suction. In contrast, water rinsing was the preferable method during dental prosthesis try-ins. It is crucial to note that dental personnel should be cautious and adhere to the universal precaution concept throughout these procedures because some patients may not inform the dentist of an infectious disease that they have.

Protective grinding devices are frequently employed when grinding a substantial quantity of acrylic resin, whereas minor grinding and adjustment are typically managed with air blowing. Theoretically, smaller particle sizes cause greater health hazards. Larger particles with at least a 5-μm diameter tend to quickly settle on surfaces, however, smaller particles with less than a 5-μm diameter can remain in the air for up to 3 h, making them susceptible to inhalation and entry into the lower respiratory tract [9], potentially causing respiratory system damage [1]. Consistent with this concept, a previous study found that grinding ceramic materials generated a higher percentage of particles with less than a 5-μm diameter compared with acrylic resin and vitallium. The toxicity of porcelain dust exceeded that of vitallium dust or PMMA dust, with cytotoxicity increasing with longer exposure times and higher concentrations [7]. Microparticles breakdown from resin-based composites also results in monomer elution that acts as an environmental pollutant [13]. Particles originate from the dental material itself but also from the grinding bur abrasion during finishing and polishing procedures [16]. Therefore, dentists should increase their awareness and concern regarding microbial spread and the toxicity of ground particles, including when performing small amounts of prosthesis grinding and polishing.

Despite the increased use of protective grinding devices and self-protection methods following the COVID-19 pandemic, approximately one-third of the surveyed dentists did not or rarely utilize protective grinding devices. Among the non-users, the reasons for not utilizing are attributed to device unavailability and the absence of preferred and user-friendly designs. Dental assistants typically follow the directives of their supervising dentists when it comes to the use of protective grinding device. This compliance may be due to various issues associated with the available protective devices, including challenges in controlling the dust dispersion and the non-ergonomic design that affects device inconvenience. Some dentists employ room cleaning, air filter usage, and personal protection equipment to minimize exposure to ground particles. However, it is imperative to highlight that the patients without access to protective devices, relying solely on the air filters and surgical drapes, might have insufficient protection against inhaling grinding particles or microbial transmission. Most dentists prefer to perform prosthesis grinding in positions adjacent to the patients, which can lead to easier inhalation of dust and the potential spread of microbes. Dust particles can also adhere to gloves during grinding, raising the possibility of their transfer into the patient’s oral cavity and posing a toxicity risk. Additionally, not cleaning the protective grinding devices or the treatment room floor after each case can result in microbial residue, contributing to the potential spread of infection [10]. Thus, the use of a protective grinding device is crucial for the dental personnel and patients’ health safety. However, the convenience and clinical efficiency should be considered when devising methods or protocols to prevent the dissemination of particles and microbes during routine clinical finishing and polishing procedures.

This study highlights the importance of educating and training dental professionals in the implementation of effective strategies to minimize dust particle dispersion during grinding and polishing the dental prostheses and equipment. Raising awareness and improving practices related to dust control is crucial for the occupational health and safety of the dentists and dental assistants and the patients who do not have protective devices. Initiating this education and standard protocol development should commence during dental school. This is essential because, in addition to the COVID-19 context, there are other aerosol-transmissible diseases, such as tuberculosis and pneumonia that can potentially be transmitted during dental procedures. Dental personal must adhere to the universal precaution concept throughout the procedures, considering that patients may not report that they have an infectious disease.

The present study has some limitations. There may be a non-response bias among individuals with a negative attitude towards protective grinding devices or those who infrequently use a protective grinding device. This survey did not collect information on the type of disinfectants used and their duration, which limits our ability to determine whether the disinfection practices were sufficient. Furthermore, the present survey did not comprehensively identify the protocols for both intraoral and extraoral devices used for controlling particle dispersion, which is necessary to ensure the protection of dental personnel during grinding and polishing procedures. To address these limitations, further research and collaboration with dental professionals are recommended to develop local standard guidelines and protocols for controlling infection spreading and dust particle dispersion. Moreover, there is a need for the continued development of protective grinding devices with user-friendly and ergonomic designs to encourage their use among dentists.

## Conclusion

Many dentists and dental assistants have increased their use of protective grinding devices during grinding and polishing dental prostheses and appliances. However, a considerable number reported infrequent use. This issue is of concern because the dust particles generated during dental prosthesis and appliance grinding and polishing can pose health hazard, particularly for dental staff and patients lacking protective wear during clinical procedures. Establishing a protocol for the regular use of protective grinding devices is imperative. Additionally, the invention of a user-friendly device is crucial, ensuring its consistent usage irrespective of the risk of disease transmission or the quantity and size of dust particle.

## Data Availability

Dataset generated during the current study is available upon request to the corresponding authors.
